# Longitudinal association between maternal cardiovascular health in pregnancy and child birth outcomes

**DOI:** 10.1038/s41598-024-66029-6

**Published:** 2024-07-04

**Authors:** Guangzhuang Jing, Qian Wei, Jiaojiao Zou, Yunhui Zhang, Huijing Shi, Xiang Gao

**Affiliations:** 1 Department of Maternal, Child and Adolescent Health, School of Public Health, Fudan University, Dong’an Road, 130, Shanghai, 200032 China; 2 Department of Nutrition and Food Hygiene, School of Public Health, Institute of Nutrition, Fudan University, Dong’an Road, 130, Shanghai, 200032 China; 3 Department of Environment Health, School of Public Health, Fudan University, Dong’an Road, 130, Shanghai, 200032 China

**Keywords:** Maternal cardiovascular health, Pregnant women, Life’s essential 8, Birth weight, Adverse birth outcome, Cohort study, Epidemiology, Risk factors, Paediatric research, Epidemiology, Diabetes, Metabolic syndrome, Obesity

## Abstract

The American Heart Association has updated its definition of cardiovascular health (CVH) with a new framework known as Life's Essential 8 (LE8). Although gestational CVH assessment has been recommended, its significance based on LE8 for birth outcomes is unknown. We thus evaluated the status of gestational CVH based on LE8 in 3036 pregnant women of the Shanghai Maternal-Child Pairs Cohort and the population of China Maternal Nutrition and Health Sciences Survey, and also examined the association between gestational CVH and child birth outcomes. We found that only a small proportion (12.84%) had high CVH, while 1.98% had low CVH in this cohort study. In adjusted models, a 10-point increase in the gestational CVH score, indicating a more favorable score, was associated with lower neonatal size such as birth weight (β: − 37.05 [95% confidence interval: − 52.93, − 21.16]), birth length (− 0.12[− 0.22, − 0.01]), weight-for-height z-score (− 0.07[− 0.12, − 0.03]), body mass index z-score (− 0.09 [− 0.13, − 0.04]), length-for-age Z-score (− 0.03 [− 0.06, − 0.01]), and weight-for-age z-score (− 0.08 [− 0.12, − 0.05]). Also, a 10-point increase in the gestational CVH score was associated with the lower risk of large for gestational age (LGA) (0.82 [0.73, 0.92]) and macrosomia infant (0.75 [0.64, 0.88]). CVH categories showed similar results. That is, better maternal CVH status in pregnancy was associated with lower neonatal size and lower risks for LGA and macrosomia in newborns.

## Introduction

The gestational period has been increasingly acknowledged as a critical time for the lifelong health of offspring after birth, and physiological adaptations occur to promote the optimal growth and development of the fetus in this period^1,2^. The theory of the Developmental Origins of Health and Disease suggests that poor intrauterine exposure during pregnancy may affect the birth outcome and have implications for long-term adult health^3^. Previous independent studies have consistently demonstrated a significant association between several cardiovascular risk factors during pregnancy, including obesity^4^, diabetes^5^, hypertension^6^, dyslipidemia^7^, sleep disorders^8^, and the higher risk of adverse birth outcomes (ABOs), such as macrosomia and large for gestational age (LGA) infant. However, during pregnancy, the combination of risk factors below the clinical diagnostic threshold is more common and may have a greater impact on maternal and infant health^9^.

To enhance the assessment of cardiovascular health (CVH) across the lifespan, the American Heart Association (AHA) introduced the ‘Life's Simple 7’ (LS7) construct in 2010^10^. And, in 2022, the AHA updated the construct into ‘Life’s Essential 8’ (LE8), which incorporates eight metrics into the assessment of CVH, including body mass index (BMI), blood lipids, blood glucose, blood pressure, sleep health, diet, physical activity (PA), and nicotine exposure^11^. The LE8 construct can calculate a composite CVH score and differentiate between high and low CVH as well as conducting more nuanced evaluations in both research and clinical settings^11^. In the assessment of nonpregnant adult CVH, improved CVH was strongly linked to various positive health outcomes, such as enhanced longevity^12^ and a reduced risk of cardiovascular diseases^12–14^. Pregnancy is commonly recognized as a crucial window into future CVH in pregnant women and offspring, and the AHA and the American College of Obstetricians and Gynecologists (ACOG) also strongly recommended optimizing CVH during pregnancy to improve health across the life course and for subsequent generations^2,15,16^. However, there is a scarcity of literature on CVH during pregnancy. Existing literature suggests that maintaining optimal CVH during pregnancy, assessed by five metrics of LS7 (BMI, blood lipids, blood glucose, blood pressure, and nicotine exposure), may mitigate maternal cardiovascular disease, ABOs^17^, and the risk of impaired CVH in offspring during adolescence^18^.

In addition to the traditional metabolic factors, behavioral factors such as sleep health^19,20^, diet^21^, and physical activity^22^ may also play a crucial role in evaluating CVH during pregnancy^23^ and influencing birth outcomes. Studies have shown that maintaining a healthy diet during pregnancy, such as following the Dietary Approaches to Stop Hypertension (DASH) diet, is positively associated with higher birth weight of offspring^24^. Plus, poor sleep quality in late pregnancy is linked to an increased risk of preterm birth and small-for-gestational-age (SGA) infants^25^, while effective exercise during pregnancy is associated with a reduced risk of macrosomia^26^. Additionally, previous studies on the assessment of CVH during pregnancy have focused on metabolic factors^17,27^, ignoring the role of behavioral factors (e.g., sleep, physical activity, and diet) in the assessment of CVH during pregnancy. Moreover, since the AHA updated the CVH construct in 2022, no study has yet examined the impact of gestational CVH, using the new LE8 construct, on fetal birth outcomes.

Therefore, the objective of this study was to investigate the association of maternal CVH after 20 weeks of gestation measured by the LE8 construct and newborn birth outcomes in a Chinese cohort of pregnant women.

## Materials and methods

### Study design and participants

We obtained data from two populations in China: Shanghai Maternal-Child Pairs Cohort (Shanghai MCPC) and the China Maternal Nutrition and Health Sciences Survey (CMNHSS). The Shanghai MCPC was based on two regional maternity hospitals in Shanghai of China, described in detail previously^28^, and the data collection for this cohort took place between April 2016 and April 2018. The CMNHSS was initiated in January 2021 as a cross-sectional survey aiming to comprehensively evaluate various aspects of maternal nutrition and health throughout the entire pregnancy (including dietary status, physical examination, metabolic disease, lifestyle and mental health), and to track the birth information for infants. For this study, we selected pregnant women from four survey sites in CMNHSS who were recruited between 2021 and 2023: Obstetrics and Gynecology Hospital of Fudan University, Shanghai; Shanghai Seventh People’s Hospital; Fujian Maternity and Child Health Hospital; Fuqing Maternal and Child Health Hospital. We pooled the data from these two populations to conduct our analysis.

**Figure 1:**
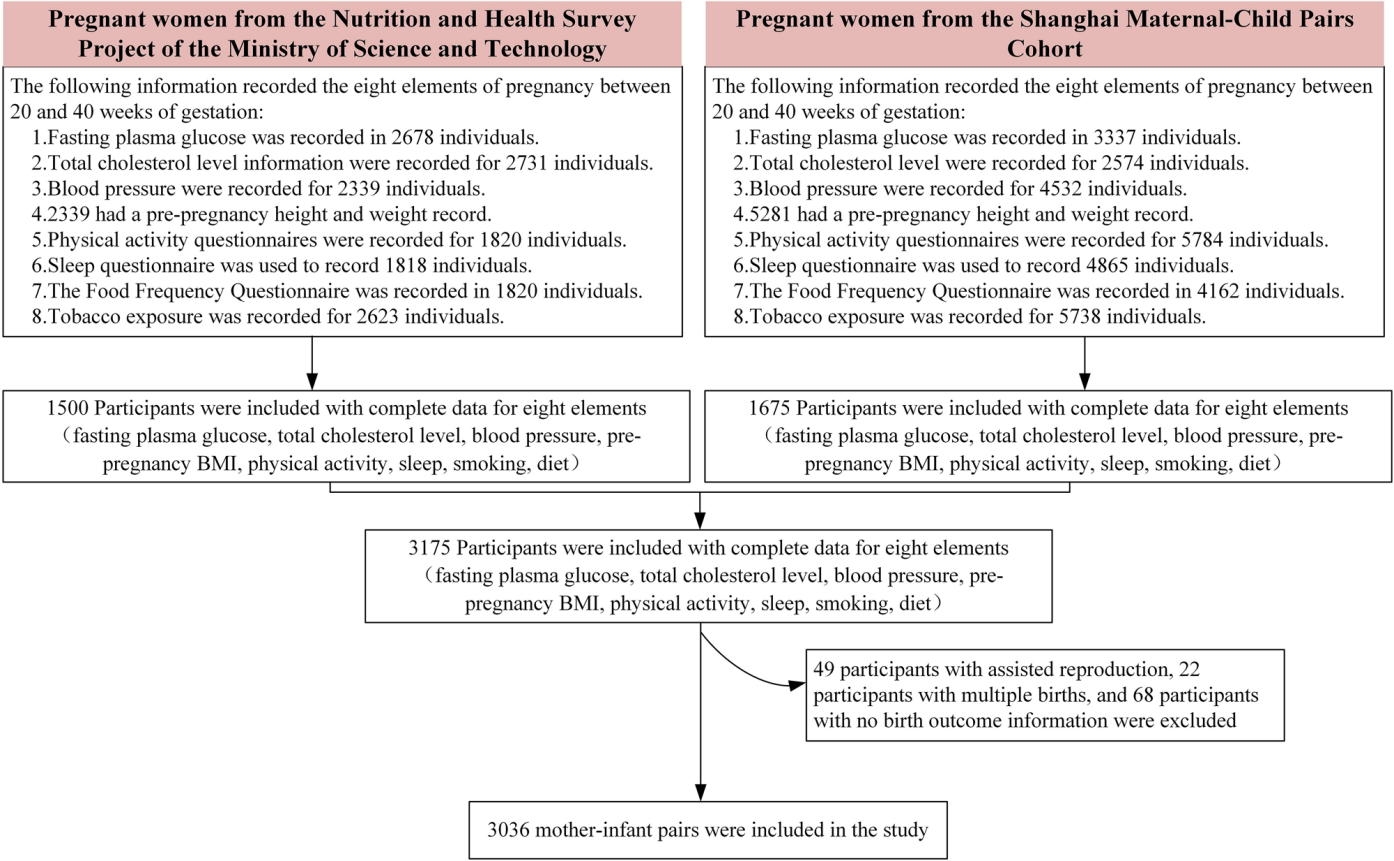
Flowchart of study participants.

A total of 3175 pregnant women who had comprehensive information of the LE8 after 20 weeks of gestation were included. To maintain a homogeneous study population, pregnant women who underwent assisted reproduction and those who had multiple births or were lack of birth data were excluded from the analysis. As a result, a final sample of 3036 mother–child dyads remained for the analysis, as depicted in Fig. 1.

### Assessment of gestational cardiovascular health

We assessed the maternal CVH characteristics based on the LE8 construct using examination data collected between 20 and 40 gestational weeks. Data from multiple physical examinations were available during pregnancy. In order to minimize variation in the pregnancy data for each metrics, we first matched the LE8 data from 24 to 32 weeks based on the gestational weeks range from previous literature^17^. Subsequently, if no data were available in this range, we chose the data that were closest to that gestational week range. The median gestational age included in the final CVH was 29.3 (see Supplementary Table S2).

Based on the AHA’s LE8^11^ and previous gestational CVH study^2,17,18,23^, maternal CVH after 20 weeks of gestation in our study was determined by combining eight ‘modified’ CVH metrics: pre-pregnancy BMI, blood pressure (BP), total cholesterol level (TC), fasting plasma glucose (FPG), sleep time, diet quality (assessed by the Dietary Approaches to Stop Hypertension eating pattern), physical activity (PA), and smoking. The definitions and scoring for these metrics have been provided in Supplementary Methods and Table S1. Each metric was scored on a scale of 0–100 points; then, the overall CVH score was calculated as the mean of the eight metrics, ranging from 0 (the lowest) to 100 (the highest). Considering that CVH categories have more clinical significance in predicting long-term health, the gestational total CVH was categorized as high (≥ 80), moderate (50–79), and low CVH (≤ 50) and the single CVH metric was categorized as ideal (≥ 80), intermediate (50–79), and poor (≤ 50), based on the LE8 construct^11^.

### Birth physical growth and adverse birth outcomes

Birth weight, length, 1-min Apgar score, and newborn sex were extracted from electronic medical records. Gestational age was determined using both last menstrual period and ultrasound dating. To assess the nutritional status of the newborns, we calculated Z-scores for the following indices: weight-for-age Z-score (WAZ), weight-for-height Z-score (WHZ), length-for-age Z-score (LAZ), and BMI-for-age Z-score (BMIZ) using WHO Anthro version 3.2.2^29^, which all have been specifically adapted for the Chinese population^30^. In our study, the birth weight, length, and the aforementioned four Z-scores were employed as pivotal indicators of birth physical growth.

In this study, we focused on a range of ABOs, namely preterm birth, small for gestational age (SGA), large for gestational age (LGA), low birth weight (LBW), macrosomia, and 1-min Apgar score. Specifically, the preterm birth was defined as newborns with a gestational age of less than 37 weeks. We categorized infants as SGA if their birth weight fell below the 10th percentile, while infants with a birth weight above the 90th percentile were classified as LGA, based on birth weight reference percentiles specific to the Chinese population^31^. Macrosomia was defined as a birth weight exceeding 4000 g, while a birth weight below 2500 g was considered indicative of a LBW infant. The Apgar score at 1 min was used as an indicator to assess neonatal asphyxia, and the score below 7 was considered indicative of neonatal asphyxia^32^.

### Covariates

Based on previous research^17,33^ and directed acyclic graphs (Fig. S1), the following variables were considered potential confounders: maternal age at delivery (in years, continuous); parity (primiparous/non-primiparous); maternal education level (middle school and below/high school/junior college/college and above); average income per month (< RMB (Renminbi) < 50,000/RMB 50,000–100,000/RMB > 10,000); gestational weight gain (GWG) (insufficient weight gain/suitable weight gain/excessive weight gain, based on the pre-pregnancy BMI referring to the IOM recommended ranges^34^); maternal alcohol use before pregnancy (yes/no), gestational age (weeks, continuous), infant sex (male/female), and paternal age (in years, continuous).

Maternal characteristics, such as maternal age at delivery, parity, pre-pregnancy weight, height, weight before delivery, gestational age, and infant sex, were extracted from the medical records. Data of maternal education level, average income per month, maternal alcohol use before pregnancy, paternal age were collected by questionnaire.

### Statistical analyses

Statistical analyses were conducted using R 3.5.3 (The R Project for Statistical Computing, Vienna, Austria). The significance level was set at 0.05. The percentage of missing values for confounding factor covariates ranged from 0.91% (maternal age) to 11.84% (GWG). Missing covariates were imputed using multiple imputation with 20 imputed datasets. The chi-square test or analysis of variance (ANOVA) was used to compare the distributions of participant characteristics, and the Cochran-Armitage Trend Test was used to compare the liner trend between the maternal CVH categories and the rates of ABOs. A restricted cubic spline adjusted regression model with four knots (5th, 35th, 65th, and 95th percentiles) was used to evaluate the linearity assumption of the relationship between gestational CVH and birth-related indicators, with 50 points of CVH score as the reference.

In the primary analysis, multivariable linear regression was used to estimate the regression coefficients maternal gestational CVH for birth physical growth including birth weight, length, Z-scores, and 1-min Apgar score. Since the prevalence of several ABOs was relatively low, the relative risk of gestational CVH on ABOs was approximated using the odds ratios from logistic regression. Specifically, binary logistic regression was used to assess the association between gestational CVH and binary outcomes (preterm birth and 1-min Apgar score < 7). Additionally, multinomial logistic regression was employed to estimate the relationship between maternal CVH and multiple ABOs (SGA, LGA, LBW, and macrosomia). All above analyses were adjusted for maternal age, maternal education, maternal parity, average monthly income, maternal alcohol use before pregnancy, GWG, paternal age, gestational age, and infant sex. And in the secondary analysis, we examined the associations of individual CVH metrics (ideal versus non-ideal [combined intermediate/poor] levels) with birth outcomes, adjusting for mentioned covariates of the primary analysis and the levels (ideal versus non-ideal) of the other CVH metrics, to determine whether the overall CVH associations were influenced by any single metric.

Besides, we conducted two separate sensitivity analyses for gestational CVH and ABOs associations. First, given the variability in assessing gestational CVH between weeks 20 and 40 in the primary analysis, a sensitivity analysis of LE8 indicator data from 868 cases during weeks 24 to 32 was performed to confirm the robustness of the main findings. Second, we replaced pre-pregnancy BMI with BMI measured between 24 to 32 weeks of gestation as the BMI metric for assessing gestational CVH, based on evidence suggesting that gestational BMI is appropriate for this purpose^2^. Following prior studies, we assigned scores to gestational BMI at 24–32 weeks: 100 points for values below 28.4, 50 points for values between 28.5 and 32.9, and 0 points for values above^18^.

### Ethics approval and consent to participate

All procedures of this study were approved by the Institutional Review Board of Fudan University (IRB number 2016–04-0587 and IRB number 2016–04-0587-EX) and the Medical Ethics Committee of Tongji Medical College, Huazhong University of Science and Technology (ethical approval number: [2021] Lun Shen Zi (S092) No). Written informed consent was obtained from all participants. We also confirmed that all research was performed in accordance with Declaration of Helsinki.

## Results

### Participant characteristics

**Figure 2:**
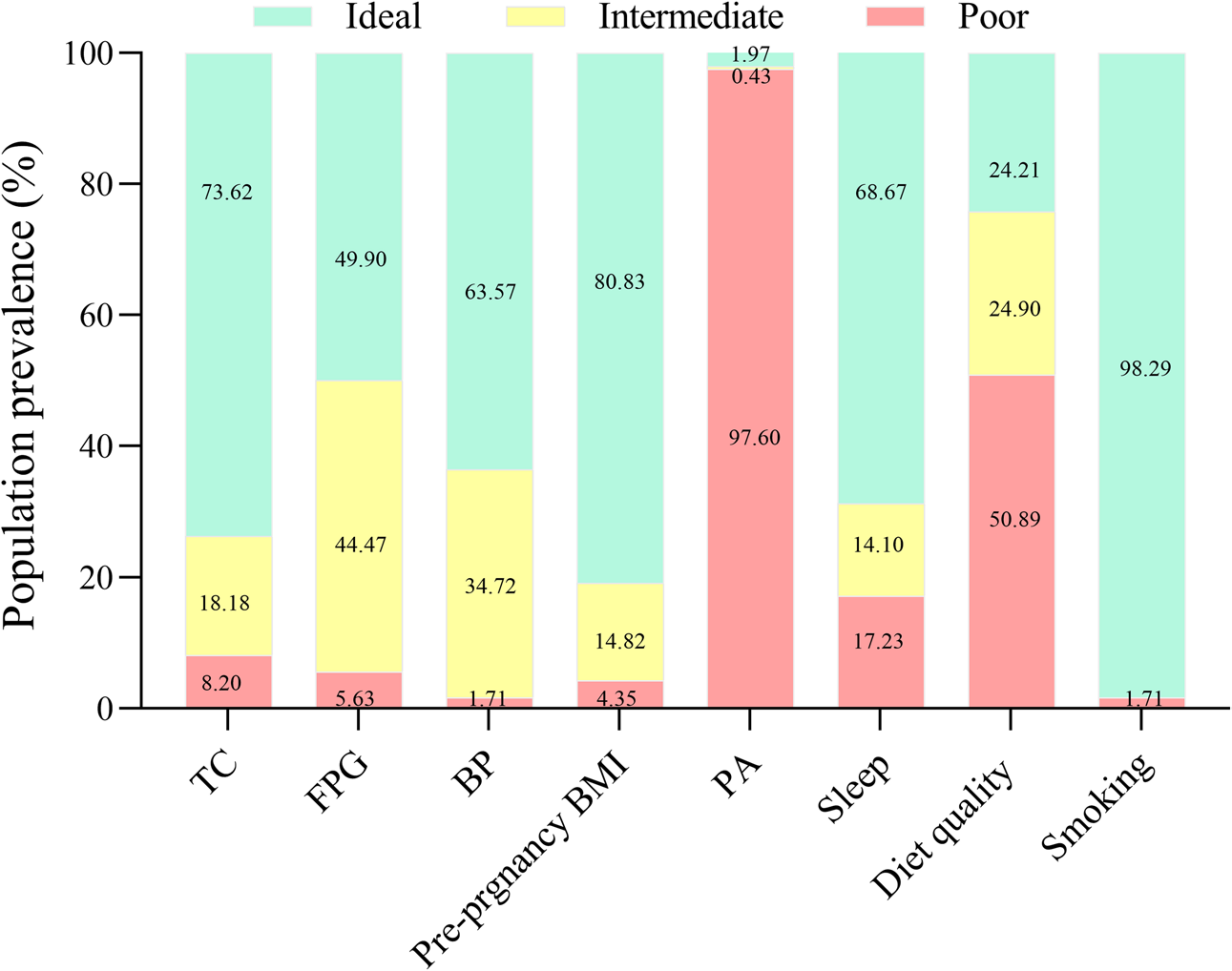
Status of individual cardiovascular health metrics among pregnant women. *FPG* fasting plasma glucose, *TC* total cholesterol, *BP* blood pressure, *PA* physical activity.

A total of 3036 pregnant women aged 18–47 years have been included to assess CVH during pregnancy. The average score of geatational CVH is 69.87 (SD 8.71) (Table S2). Only 12.84% (n = 390) pregnant women had high CVH, while 1.98% (n = 60) had low CVH (Table 1). Among the eight metrics of CVH during pregnancy, smoking, pre-pregnancy BMI, and TC were the most frequently prevalent ideal metrics, whereas the PA, diet quality, and FPG were the least frequently prevalent ideal metrics (Fig. 2).

**Table 1:** The distribution of characteristics of participants in different maternal CVH categories [Mean ± S/N (%)].

Characteristics	Overall	Low-CVH	Moderate-CVH	High-CVH	*P* value
n	3036	60 (1.98)	2586 (85.18)	390 (12.84)	
Maternal characteristics					
Maternal age (y)					
< 25	437 (14.39)	6 (10.00)	380 (14.69)	51 (13.08)	0.359
25–30	1364 (44.93)	28 (46.67)	1149 (44.43)	187 (47.95)	
30–35	911 (30.01)	15 (25.00)	784 (30.32)	112 (28.72)	
≥ 35	324 (10.67)	11 (18.33)	273 (10.56)	40 (10.26)	
Maternal education					
Middle school and below	473 (15.58)	13 (21.67)	398 (15.39)	62 (15.90)	0.017
High school	564 (18.58)	19 (31.67)	474 (18.33)	71 (18.21)	
Junior college	799 (26.32)	13 (21.67)	699 (27.03)	87 (22.31)	
College and above	1200 (39.53)	15 (25.00)	1015 (39.25)	170 (43.59)	
Income (RMB)					
< 50,000	1153 (37.98)	36 (60.00)	1005 (38.86)	112 (28.72)	0.001
50,000–100,000	1236 (40.71)	17 (28.33)	1073 (41.49)	146 (37.44)	
≥ 10,000	647 (21.31)	7 (11.67)	508 (19.64)	132 (33.85)	
Parity					
Primiparous	1775 (58.47)	27 (45.00)	1508 (58.31)	240 (61.54)	0.049
Non-primiparous	1261 (41.53)	33 (55.00)	1078 (41.69)	150 (38.46)	
Alcohol consumption before pregnancy					
Yes	507 (16.70)	6 (10.00)	437 (16.90)	64 (16.41)	0.362
No	2529 (83.30)	54 (90.00)	2149 (83.10)	326 (83.59)	
GWG					
Insufficient weight gain	654 (21.54)	8 (13.33)	575 (22.24)	71 (18.21)	0.007
Suitable weight gain	1162 (38.27)	24 (40.00)	959 (37.08)	179 (45.90)	
Excessive weight gain	1220 (40.18)	28 (46.67)	1052 (40.68)	140 (35.90)	
Paternal characteristics					
Paternal age (y)					
< 25	321 (10.57)	6 (10.00)	273 (10.56)	42 (10.77)	0.847
25–30	1243 (40.94)	21 (35.00)	1059 (40.95)	163 (41.79)	
30–35	977 (32.18)	19 (31.67)	837 (32.37)	121 (31.03)	
≥ 35	495 (16.30)	14 (23.33)	417 (16.13)	64 (16.41)	
Infant characteristics					
Infant sex					
Boys	1554 (51.19)	26 (43.33)	1335 (51.62)	193 (49.49)	0.345
Girls	1482 (48.81)	34 (56.67)	1251 (48.38)	197 (50.51)	
Preterm birth					
No	2943 (96.94)	57 (95.00)	2509 (97.02)	377 (96.67)	0.632
Yes	93 (3.06)	3 (5.00)	77 (2.98)	13 (3.33)	
Gestational age classification					
SGA	207 (6.82)	3 (5.00)	173 (6.69)	31 (7.95)	0.043
AGA	2336 (76.94)	39 (65.00)	1995 (77.15)	302 (77.44)	
LGA	493 (16.24)	18 (30.00)	418 (16.16)	57 (14.62)	
Birth weight classification					
LBW	71 (2.34)	3 (5.00)	58 (2.24)	10 (2.56)	0.127
Normal weight infant	2770 (91.24)	49 (81.67)	2365 (91.45)	356 (91.28)	
Macrosomia	195 (6.42)	8 (13.33)	163 (6.30)	24 (6.15)	
Birth weight (g), mean (SD)	3325.42 (428.32)	3478.92 (525.91)	3328.07 (425.75)	3284.23 (423.62)	0.003
Birth length (cm), mean (SD)	49.56 (2.59)	50.03 (1.34)	49.56 (2.38)	49.45 (3.81)	0.255
1-min Apgar score*, mean (SD)	9.825 (0.549)	9.838 (0.442)	9.823 (0.556)	9.842 (0.513)	0.901
1-min Apgar* score < 7 (%)	14 (0.88)	0 (0.00)	13 (0.94)	1 (0.58)	0.758

*GWG* gestational weight gain, *SGA* Small for gestational age, *LGA* Large for gestational age, *LBW* low birth weight.

*The sample size was 1595.

Table 1 displays the distribution of demographic characteristics of pregnant women, husband, and infants across different categories of gestational CVH. It was observed that pregnant women with higher CVH were more educated, had higher incomes, exhibited more appropriate GWG, and were more likely to be nulliparous (*P* < 0.05). Furthermore, it was also noted that among the 3,036 mother-newborn dyads, mothers with high CVH exhibited lower birth weight (*P* < 0.05).

### Primary analysis: associations of gestational CVH with birth physical growth and adverse birth outcomes

#### Gestational CVH and birth physical growth

In the adjusted models, a 10-point increase in the gestational CVH score, indicating a more favorable score, was associated with lower birth weight (β: − 37.05, 95% CI [− 52.93, − 21.16]), birth length (β: − 0.12, 95% CI [− 0.22, − 0.01]), WHZ (β: − 0.07, 95% CI [− 0.12, − 0.03]), LAZ (β: − 0.03, 95% CI [− 0.06, − 0.01]), BMIZ (β: − 0.09, 95% CI [− 0.13, − 0.04]), and WAZ (β: − 0.08, 95% CI [− 0.12, − 0.05]) (Table 2). Furthermore, maternal CVH categories also demonstrated associations with birth physical growth. Specifically, both moderate and high CVH during pregnancy were correlated with lower birth weight (moderate: β =  − 161.68, 95% CI [− 260.47, − 62.89]; high: β =  − 183.74, 95% CI [− 288.85, − 78.63]), birth length (high: β =  − 0.75, 95% CI [− 1.44, − 0.06]), LAZ (moderate: β =  − 0.29, 95% CI [− 0.45, − 0.12]; high: β =  − 0.25, 95% CI [− 0.43, − 0.08]), BMIZ (high: β =  − 0.34, 95% CI [− 0.63, − 0.05]), and WAZ (moderate: β =  − 0.32, 95% CI [− 0.53, − 0.11]; high: β =  − 0.37, 95% CI [− 0.59, − 0.15]), compared to low CVH (Table 2). No association was observed between gestational CVH and the 1-min Apgar score in newborns.

**Table 2:** Adjusted association of maternal gestational CVH score and categories with birth physical growth by multiple linear regression.

Gestational CVH exposure	β (95% CI) for birth physical growth	*P* value
CVH score (per 10 points higher [more favorable])		
Birth weight (g)	** − 37.05 (− 52.93, − 21.16)**	** < 0.001**^**a**^
Birth length (cm)	** − 0.12 (− 0.22, − 0.01)**	**0.027**^**a**^
WHZ	** − 0.07 (− 0.12, − 0.03)**	**0.002**^**a**^
LAZ	** − 0.03 (− 0.06, − 0.01)**	**0.013**^**a**^
BMIZ	** − 0.09 (− 0.13, − 0.04)**	** < 0.001**^**a**^
WAZ	** − 0.08 (− 0.12, − 0.05)**	** < 0.001**^**a**^
Apgar score at 1 min*	0.00 (− 0.03, 0.03)	0.886
Moderate-CVH (vs low-CVH)		
Birth weight (g)	** − 161.68 (− 260.47, − 62.89)**	**0.001**^**a**^
Birth length (cm)	− 0.63 (− 1.28, 0.01)	0.055
WHZ	− 0.13 (− 0.42, 0.15)	0.351
LAZ	** − 0.29 (− 0.45, − 0.12)**	** < 0.001**^**a**^
BMIZ	− 0.24 (− 0.51, 0.03)	0.084
WAZ	** − 0.32 (− 0.53, − 0.11)**	**0.003**^**a**^
Apgar score at 1 min*	0.00 (− 0.18, 0.18)	0.990
High-CVH (vs low-CVH)		
Birth weight (g)	** − 183.74 (− 288.85, − 78.63)**	** < 0.001**^**a**^
Birth length (cm)	** − 0.75 (− 1.44, − 0.06)**	**0.033**^**a**^
WHZ	− 0.25 (− 0.55, 0.05)	0.098
LAZ	** − 0.25 (− 0.43, − 0.08)**	**0.005**^**a**^
BMIZ	** − 0.34 (− 0.63, − 0.05)**	**0.020**^**a**^
WAZ	** − 0.37 (− 0.59, − 0.15)**	**0.001**^**a**^
Apgar score at 1 min*	0.03 (− 0.17,0.23)	0.764

The adjusted model covariates include maternal age, maternal education level, maternal parity, average income per month, maternal alcohol use before pregnancy, gestational weight gain, paternal age, gestational age, and infant sex.

*The sample size was 1595.

^a^A false discovery rate less than 0.05, the bolded effect sizes indicate statistical significance (*P* < 0.05).

#### Gestational CVH and adverse birth outcomes

**Figure 3:**
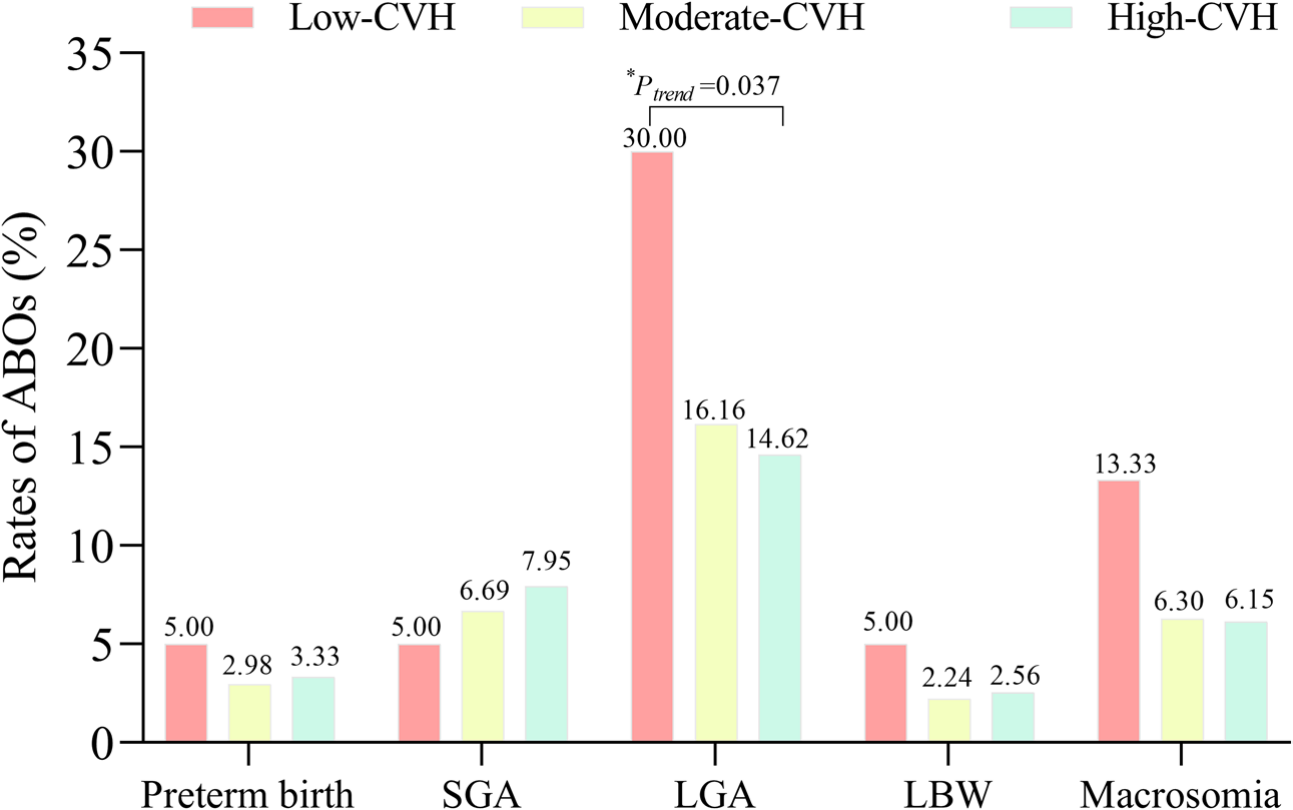
Rates of Adverse Birth Outcomes by Gestational CVH categories. *The star indicates a linear trend between different maternal CVH group and the rates of adverse birth outcomes by the Cochran-Armitage Trend Test. (*P*_*trend*_ < 0.05). *CVH* Cardiovascular Health, *ABOs* adverse birth outcomes, *SGA* small for gestational age, *LGA* Large for gestational age, *LBW* low birth weight.

The rates of ABOs by gestational CVH categories were depicted in Fig. 3. The trend chi-squared test revealed a linear association between maternal CVH categories during pregnancy and the rates of LGA (*P* < 0.05).

**Figure 4:**
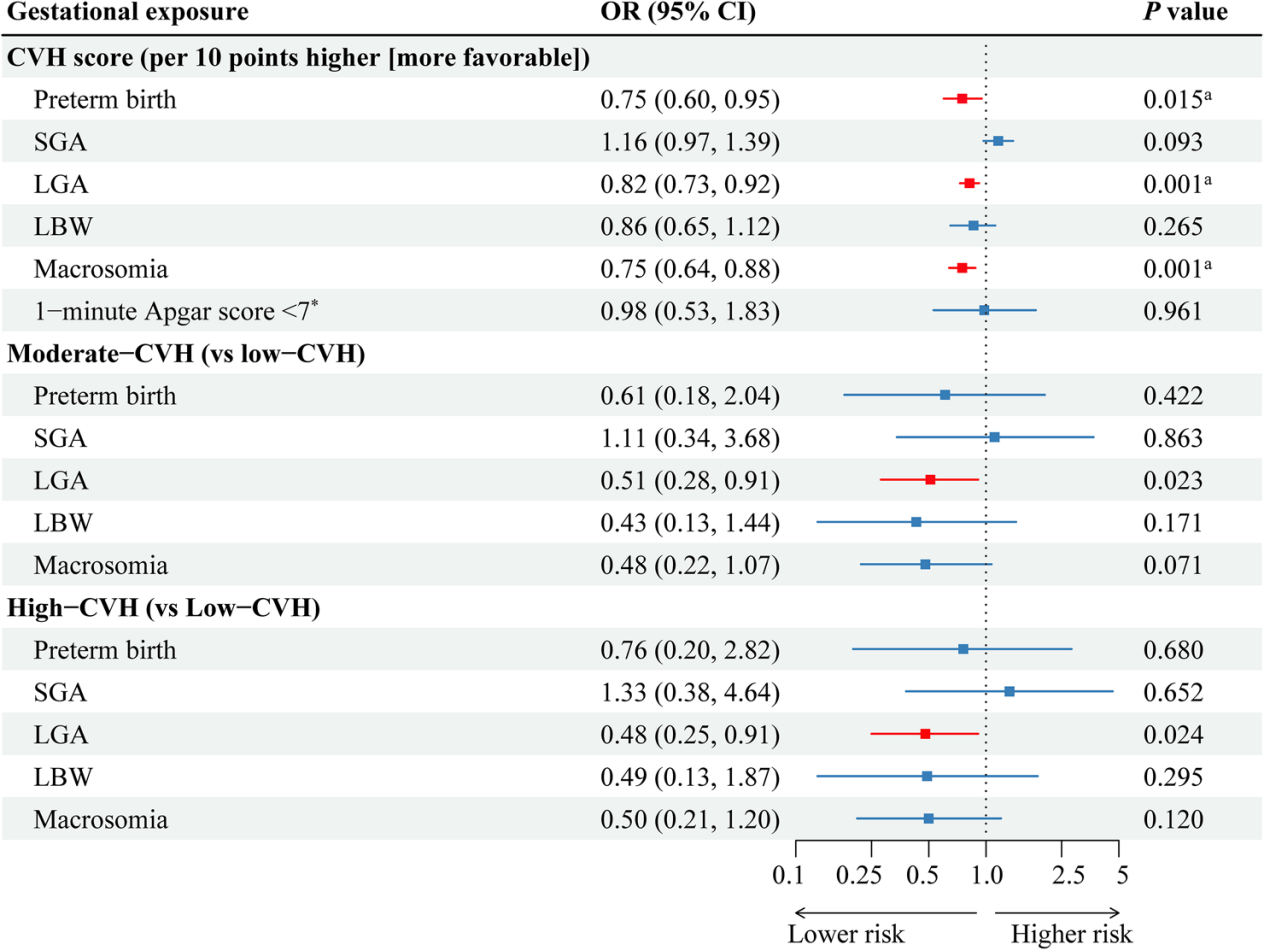
Adjusted association of maternal gestational CVH score and categories with adverse birth outcomes. The adjusted model covariates include maternal age, maternal education level, maternal parity, average income per month, maternal alcohol use before pregnancy, GWG, paternal age, and infant sex. The red squares represent statistical significance for rates of adverse birth outcomes among different maternal CVH exposure. *The sample size was 1595. ^a^A false discovery rate less than 0.05. *CVH* Cardiovascular Health, *SGA* small for gestational age, *LGA* Large for gestational age, *LBW* low birth weight.

In the adjusted models, a 10-point increase in gestational CVH score was significantly associated with lower risks of preterm birth (OR: 0.75, 95% CI [0.60, 0.95]), LGA (OR: 0.82, 95% CI [0.73, 0.92]), and macrosomia (OR: 0.75, 95% CI [0.64, 0.88]) (Fig. 4). Similarly, maternal CVH categories were also associated with ABOs, as illustrated in Fig. 4. Specifically, compared to low maternal CVH, moderate and high maternal CVH were significantly associated with lower risks of large for LGA infants, with odds ratios (OR) of 0.51 (95% CI: [0.28, 0.91]) and 0.48 (95% CI [0.25, 0.91]), respectively.

### Secondary analysis: associations of single CVH metrics with adverse birth outcomes

The gestational ideal FPG and pre-pregnancy BMI metrics (compared to non-ideal metrics) were independently and significantly associated with the lower risks of LGA (FPG: OR = 0.74, 95% CI [0.60, 0.91]; pre-pregnancy BMI: OR = 0.55, 95% CI [0.44, 0.69]) and macrosomia (FPG: OR = 0.62, 95% CI [0.45, 0.84]; pre-pregnancy BMI: OR = 0.59, 95% CI [0.43, 0.83]). Additionally, the gestational ideal TC metric (compared to non-ideal metrics) was independently and significantly associated with the lower risks of preterm birth (OR = 0.56, 95% CI: [0.36, 0.87]. However, the gestational ideal FPG and pre-pregnancy BMI metrics (compared to non-ideal metrics) were associated with an increased risk of SGA (FPG: OR = 1.41, 95% CI [1.04, 1.90]; pre-pregnancy BMI: OR = 1.66, 95% CI [1.03, 2.66]) (Fig. S2).

### Sensitivity analyses

When we reevaluated the CVH of 868 pregnant women at 24–32 weeks, the mean CVH score was 69.93 (SD 8.62). The distribution of CVH categories was as follows: 12.79% (N = 111) high, 85.71% (N = 744) moderate, and 1.50% (N = 13) low. The associations of maternal gestational CVH at 24–32 weeks with offspring birth physical growth and ABOs were similar to those in the primary analysis (Table S3 and Fig. S3). Specifically, better maternal gestational CVH score at 24–32 weeks was associated with lower neonatal size and lower risk of LGA and macrosomia, whereas the effect of preterm birth disappeared.

When the pre-pregnancy metric was defined by gestational BMI at 24–32 week, the mean CVH score was 70.08 (SD 8.79). Among 2445 pregnant women, the distribution of CVH categories was as follows: 13.82% (N = 338) high, 84.38% (N = 2063) moderate, and 1.80% (N = 44) poor. The gestational BMI metric categories (86.91% ideal, 11.25% intermediate, 1.84% poor) were superior to those in the primary analysis, which used pre-pregnancy metric (80.83% ideal, 14.82% intermediate, 4.35% poor). However, the associations between maternal CVH and birth physical growth, as well as ABOs, were also similar to those in the primary analysis. Specifically, better maternal gestational CVH score was associated with lower infant birth weight and lower risk of macrosomia, whereas the effect of preterm birth and LGA disappeared (Table S4 and Fig. S4).

## Discussion

In the prospective cohort that included 3,036 pregnant women, we found that only a small proportion (12.84%) had high CVH, while 1.98% had low CVH. Our study revealed that better maternal gestational CVH was associated with lower neonatal size (including birth weight, length, WHZ, LAZ, BMIZ, WAZ), and also related with lower risk of LGA and macrosomia in newborns.

To our knowledge, this study is the first to assess maternal CVH during the second and third trimesters in Chinese women using the LE8 construct. Due to the lack of unified standards for evaluating gestational CVH, evidence in this field remains controversial. A previous study assessed CVH among 406 American pregnant women across the first, second, and third trimesters using the LS7 construct^23^. The results showed that the proportions of ideal CVH in first, second and third trimesters of pregnancy were 4.4%, 8.6% and 0.3% respectively, with 60% having moderate CVH in the third trimester. Our results were slightly different from the above findings, revealing a low proportion of ideal CVH (12.85%), with the majority categorized as moderate (85.18%). This variability may result from the lack of uniformity in the gestational CVH assessment methodologies or demographic differences. However, despite the variability in CVH assessment methods, studies have consistently concluded that the prevalence of high CVH during pregnancy is not optimistic. In our study, ideal physical activity and diet were the least common among all ideal gestation CVH metrics, which is consistent with previous studies^23^. This may be due to the increased weight gain during pregnancy which prevents participation in moderate to vigorous physical activity; additionally, the stress of pregnancy causes pregnant women to reject nutritious food intake, which leads to generally low dietary scores.

Before the release of the LE8 framework, only the Hyperglycemia and Adverse Pregnancy Outcome study^17^ evaluated the association between gestational CVH and child birth outcomes, which revealed that maternal favorable CVH at 24–32 gestation, using the five CVH metrics of LS7 (including gestational BMI, total cholesterol, FPG, BP), could reduce the risk of macrosomia and LGA. Similarly, our result was consistent with the above mentioned. The CVH status consisted of modifiable risk factors that can reduce the risk of LGA by 52% if the pregnant woman is in high CVH. Notably, we found that the combined high-CVH (52%) had a greater protective effect on LGA than any of the individual metrics (FPG: 26%, pre-pregnancy BMI: 45%) by comparing the protective effects of the individual metrics on LGA. This implies that the combined CVH of pregnant women may have a comprehensive and critical impact on the prevention of excessive neonatal birth weight and it suggests a holistic approach to monitoring the CVH of pregnant women rather than focusing on a single metric solely. This perspective may provide a more effective strategy for risk management during pregnancy.

Our result highlighted that the protective effect of maternal CVH on LGA or macrosomia was likely driven by pre-pregnancy BMI and FPG, which was similar to the result of previous study that the key metrics of gestational CVH at 24–32 weeks affect LGA or macrosomia were FPG, BMI, and TC^17^. It’s hinted that the protective mechanism of maternal CVH on ABOs may be related to the metabolic mechanisms during pregnancy. Additionally, gestational PA, sleep duration and diet quality, as modifiable factors in pregnancy, we did not find any significant association of ideal gestational PA, sleep duration and diet quality with the risk of ABOs. However, previous studies suggested that gestational sleep quality indicators^27^, e.g. snoring^35^, insomnia^36^, were associated with birth outcomes, whereas sleep duration was not^37^. Dash dietary pattern during pregnancy was positively associated with physical growth indexes such as birth weight, length, and head circumference^38^, whereas physical activity was more associated with late pregnancy complications such as preeclampsia^39^. The above suggests that more detailed behavioral indicators need to be included in future CVH assessments during pregnancy.

Our study has several clinical implications. First, we suggest a simple construction of a CVH risk score for pregnant women. In pregnant women with low CVH, prescription interventions for behavioral factors can be given to prevent excessive birth weight in newborns. Second, our study also has important implications for the prevention of cardiovascular disease in offspring. Previous research has implicated that ABOs (such as preterm birth^40^, macrosomia^41,42^, and LGA^43^) as potential risk factors were associated with long-term cardiovascular metabolic diseases in pregnant women^44^ and infants^45^. This suggests that the protective effect of gestational CVH on ABOs may be a potential pathway for preventing long-term cardiovascular disease. Finally, a standardized and exacting standard for measuring CVH in pregnant women is urgently needed, along with methods for implementing CVH monitoring and risk modification.

## Strengths and limitations

Strengths of our study include cohort study design in Chinese pregnant women, comprehensive metrics of CVH, and multiple sensitivity analyses.

However, our study also has some limitations. Firstly, our inclusion of gestational CVH after the 20th week is a wide time span, which may bring certain bias. In response, we selected pregnant women from 24 to 32 weeks for sensitivity analysis, which validated the conclusions of the main analysis. In the future, if conditions permit, we will conduct a trimester-specific assessment of CVH during pregnancy. Secondly, our study was based on a natural population birth cohort and possible medical interventions were not taken into account, which may impact on the results. Thirdly, due to the data limitations, our study did not include several important ABOs, such as diabetes mellitus in the third trimester, preeclampsia and NICU in newborns. If subsequent data collection is complete, we will explore the association of pre-pregnancy CVH and the CVH at early pregnancy with preeclampsia, gestational diabetes, and NICU in newborns. Finally, there are no standardized guidelines for assessing CVH during pregnancy, and we primarily referred to established, high-quality publications. Therefore, further research concerning the CVH measurement and risk stratification specifically for pregnant women are required.

## Conclusion

In conclusion, our study found that more favorable total CVH status was associated with lower neonatal size and lower risks for LGA and macrosomia in newborns.

### Supplementary Information

Supplementary Information.

## Data Availability

The data underlying this article will be shared on reasonable request to the corresponding author.
