# Supplementary material for: Longitudinal association between maternal cardiovascular health in pregnancy and child birth outcomes
Source: Sci Rep. 2024 Jul 4;14:15355. doi: 10.1038/s41598-024-66029-6 (PMC11222450; doi:10.1038/s41598-024-66029-6)
Supplement: Supplementary file 1 — Supplementary Information. [file 41598_2024_66029_MOESM1_ESM.pdf]

Supplementary Materials

Longitudinal association between Maternal Cardiovascular Health in Pregnancy and child birth outcomes

Supplementary Methods

Table. S1 Definition and scoring approach for quantifying maternal cardiovascular health metrics, as per the American Heart Association's Life's Essential 8 construct

Table. S2 Life's Essential 8 scores of the overall population

Table. S3 The sensitivity analysis of adjusted association of maternal gestational CVH at 24-32 gestation with birth physical growth (n=868)

Table. S4 The sensitivity analysis of adjusted association between maternal gestational CVH and birth physical growth, using gestational BMI at 24-32 gestation (instead of pre-pregnancy BMI) as the BMI metric (n=2445).

Figure. S1 Directed acyclic graph depicting the relationship between maternal CVH in pregnancy and child birth outcome and relevant covariates available for this analysis

Figure. S2 Adjusted association between individual gestational cardiovascular health metrics and adverse birth outcomes

Figure. S3 The sensitivity analysis of adjusted association between maternal gestational CVH at 24-32 gestation and adverse birth outcome.

Figure. S4 The sensitivity analysis of adjusted association between maternal gestational CVH and adverse birth outcome, using gestational BMI at 24-32 gestation (instead of pre-pregnancy BMI) as the BMI metric.

Affiliated Reference:

Supplementary Methods

Gestational Cardiovascular health assessment using Life's Essential 8 metrics

Diet quality

Dietary intake during pregnancy was collected using a self-reported Food Frequency Questionnaire (FFQ), which was adapted from the China Nutrition and Health Survey and the questionnaire developed by Zhao[1]. It included 40 food items representing habitual dietary intake. This questionnaire has been widely used for assessing dietary intake during pregnancy[2]. We extracted 8 dietary components involving fruits, vegetables, nuts and legumes, whole grains, low-fat dairy, sodium, red and processed meats, sweetened beverages, and converted into DASH scores based on previous studies[3]. The Diet quality level were identified by the DASH scores (1st－24th, 25th－49th, 50th－74th, 75th－94th, and ≥ 95th percentile values, corresponding to 0, 25, 50, 80, and 100 points, respectively)[4].

Physical activity

The duration of moderate-to-vigorous physical activity during the past week in the second and third trimesters of pregnancy was assessed using the International Physical Activity Questionnaire[5]. The questionnaire comprised seven questions, requiring participants to report the frequency and average duration of sedentary activity, walking, moderate-intensity activity, and vigorous-intensity activity over the past seven days. Vigorous-intensity activities included exercises requiring effort and causing breathing to be faster than usual, such as running, swimming, and aerobics. Moderate-intensity activities referred to exercises that required effort and resulted in breathing being slightly faster than usual, such as cycling, tai chi, and ballroom dancing. In this study, the cumulative time spent in moderate and vigorous activities over the past week in the second and third trimesters was calculated.

Moderate- and vigorous-intensity Physical activity time was classified as 0, 1–29, 30–59, 60–89, 90–119, 120–149, and ≥ 150 min per week, corresponding to 0, 20, 40, 60, 80, 90, and 100 points, respectively[4,6].

Smoking

Information on self-reported nicotine exposure collected in the questionnaire was used including gestational smoking and secondhand tobacco. The nicotine exposure was classified as ‘Never smoke and no secondhand smoke exposure’, ‘No smoking, secondhand smoke exposure’, and ‘Smoke’, corresponding to 0, 80, and 100 points, respectively[4].

Sleep time

Sleep health was a new metric in LE8 goals based on the self-reported average hours of sleep per night collected by the Chinese version of Pittsburgh Sleep Quality Index[7] (PSQI) item ‘How much time you actually slept each night in the past week’. It has been validated for reliability and validity in pregnant women[8]. According to the AHA’s LE8 Construct[4], Sleep hours per night were classified as < 4, 4 to < 5, 5 to < 6 or ≥ 10, 6 - < 7, 9 - < 10, and 7 - < 9 h, corresponding to 0, 20, 40, 70, 90, and 100 points, respectively[4].

Body mass index

Considering the variability of GWG, we used pre-pregnancy BMI to represent pregnancy BMI. Pre-pregnancy BMI was calculated by dividing the weight in kilograms by the square of height in meters. Based on the guidelines for prevention and control of overweight and obesity in Chinese adults[9], the Pre-pregnant BMI levels were classified as ≥40.0, 30.0–40.0, 28.0–29.9, 24.0–27.9, and < 24 kg/m^2, corresponding to 0, 15, 30, 70, and 100 points, respectively.

Total cholesterol

Due to data limitations, TC was used as a substitute for non-high density lipoprotein cholesterol. Total cholesterol level was obtained from the medical records of the pregnant women. Based on the Hyperglycemia and Adverse Pregnancy Outcome Study[10], the TC was classified as ≥ 300, 220-300, and ≤ 220 mg/dL, corresponding to 0, 50, and 100 points, respectively.

Fasting plasma glucose

Fasting plasma glucose level was obtained from the medical records of the pregnant women. According to the textbook of obstetrics and gynecology[11], the FPG

was classified as ≥ 5.1, 4,4-5.1 mg/dL, and < 4.4 mg/dL, corresponding to 0, 60, and 100 points, respectively.

Blood pressure

Gestational blood pressure was measured by trained study doctors using calibrated instruments. Each pregnant woman was inquired to rest while seated for at least 5 minutes before the measurement. When measuring BP, repeat the measurement 1 to 2 min apart and take the average of the 2 readings and record. If the difference between the 2 readings of SBP/DBP is >5mm Hg, the measurement should be repeated for a third time and the average of the 3 readings were recorded. Based on the Chinese expert consensus on blood pressure management during pregnancy[12], gestational blood pressure levels were classified as SBP ≥ 160 mmHg or DBP ≥ 100 mmHg, 140-159 or 90-99 mmHg, 130-139 or 80-89 mmHg, 120-129/ < 80 mmHg, and < 120/80 mmHg, corresponding to 0, 25, 50, 75, and 100, respectively.

Table S.1 Definition and scoring approach for quantifying maternal cardiovascular health metrics, as per the American Heart Association's Life's Essential 8 construct

Domain	CVH metrics	Method of measurement	Quantification of CVH Metric-Pregnant women
**Health Behaviors**	**Diet quality** [4]	**Measurement:** Self-reported daily intake of a DASH-style eating pattern (The DASH food components from the FFQ questionnaire were extracted[3])	**Metric:** Fruits, Vegetables, Nuts and Legumes, Whole Grains, Low-fat Dairy, Sodium, Red and Processed Meats, Sweetened beverages.
		**Example tools for measurement:**<br>Diet quality score (populations)	**Scoring:**<br>**Points** **Quantile**<br>100 ⩾95th percentile (top/ideal diet)<br>80 75th-95th percentile<br>50 50th–74th percentile<br>25 25th–49th percentile<br>0 1st–24th percentile (bottom/least ideal quartile)
	**Physical activity**[4]	**Measurement:**<br>Self-reported minutes of moderate or vigorous PA per week	**Metric:** Minutes of moderate- (or greater) intensity activity per week.
		**Example tools for measurement:**<br>The International Physical Activity Questionnaire	**Scoring**[13]**:**<br>**Points** **Minutes**<br>100 ⩾150<br>90 120-149<br>80 90-110<br>60 60-89<br>40 30-59<br>20 1-29<br>0 0
	**Smoking**	Measurement: The tobacco use questionnaires and the indoor smoking status questionnaire for household smokers.	**Metric:** The tobacco use or secondhand smoke exposure

		**Example tools for measurement:**<br>"Have you smoked in the last month?" and "How many passive smoking days do you have more than 15 days per week?"	**Scoring:**<br>**Points** **Minutes**<br>100 Never smoker<br>80 Passive smoker<br>0 Current smoker
	**Sleep time**[4]	**Measurement:** Self-reported average hours of sleep per night	**Metric:** Average hours of sleep per night.
		**Example tools for measurement:**<br>"In recent months, how many hours of sleep do you usually get at night?"	**Scoring:**<br>**Points** **Level**<br>100 7–<9<br>90 9–<10<br>70 6–<7<br>40 5–<6 or ⩾10<br>20 4–<5<br>0 <4
**Health factors**	**Pre-pregancy Body mass index**[14]	**Measurement:**<br>Pre-pregancy Body weight (kg) divided by height squared (m^2)	**Metric:** BMI (kg/m2)
		**Example tools for measurement:**<br>Objective measurement of pre-pregancy height and weight	**Scoring:**<br>**Points** **Level**<br>100 <24<br>70 24.0–27.9<br>30 28.0–29.9<br>15 30.0–40.0<br>0 ⩾40.0
		**Measurement:** Plasma total cholesterol (TC)	**Metric:** TC / (mg/dL)

	**Blood lipids**[15]	**Example tools for measurement:**<br>Fasting or nonfasting blood sample, according to previous study[16]	**Scoring:**<br>**Points Level**<br>100 <260<br>50 260-300<br>0 ＞300
	**Blood glucose**[11]	**Measurement:** Fasting plasma glucose (FPG)	**Metric:** FPG / (mmol/L)
		**Example tools for measurement:**<br>According to the criteria of impaired fasting glucose and GDM during pregnancy.	**Scoring:**<br>**Points Level**<br>100 <4.4<br>60 4.4–5.1<br>0 ⩾5.1
	**Blood pressure**[12]	**Measurement:**<br>Appropriately measured systolic and diastolic BPs	**Metric:** Systolic and diastolic blood pressure (mmHg)
		**Example tools for measurement:**<br>Appropriately sized blood pressure cuff	**Scoring:**<br>**Points Level**<br>100 <120/<80<br>75 120-129/<80<br>50 130-139 or 80-89<br>25 140-159 or 90-99<br>0 ⩾160 or ⩾100<br>Subtract 20 points for preeclampsia and/or eclampsia

Table. S2 Life's Essential 8 scores of the overall population

CVH metrics	Gestation	Overall	Low/Poor-CVH[a]	Moderate/Intermediate-CVH[a]	High/Ideal-CVH[a]
scores	Median (IQR)	Mean (SD)	N (%)	N (%)	N (%)
CVH	29.3 (20.2, 39.1)	69.87 (8.71)	60 (1.98)	2586 (85.18)	390 (12.84)
FPG	26.1 (24.9, 27.0)	76.58 (26.95)	171 (5.63)	1350 (44.47)	1515 (49.90)
TC	34.3 (29.4, 38.9)	82.71 (31.24)	249 (8.20)	552 (18.18)	2235 (73.62)
BP	26.0 (24.0, 32.0)	86.59 (19.90)	52 (1.71)	1054 (34.72)	1930 (63.57)
Pre-pregnancy BMI	——	92.23 (18.17)	132 (4.35)	450 (14.82)	2454 (80.83)
PA	32.0 (28.0, 35.0)	2.31 (14.06)	2963 (97.60)	13 (0.43)	60 (1.97)
Sleep time	32.0 (28.0, 32.0)	83.12 (24.63)	523 (17.23)	428 (14.10)	2085 (68.67)
Diet quality	32.0 (28.0, 35.0)	39.819 (30.77)	1545 (50.89)	756 (24.90)	735 (24.21)
Smoking	32.0 (28.0, 34.0)	95.49 (14.38)	52 (1.71)	0 (0)	2984 (98.29)

The horizontal line "——" indicates that no data in this part.

[a] Gestational total CVH was categorized as high (⩾80), moderate (50-79), and low CVH (⩽50) and the single CVH metric was categorized as ideal (⩾80), intermediate (50-79), and poor (⩽50), based on the LE8 construct

Abbreviations: CVH, Cardiovascular health; FPG, fasting plasma glucose; TC, total cholesterol; BP, blood pressure; PA, physical activity.

Table. S3 The sensitivity analysis of adjusted association of maternal gestational CVH at 24-32 gestation with birth physical growth (n=868)

**Gestational CVH exposure**	**β (95% CI) for birth physical growth**	***P* value**
**CVH score (per 10 points higher [more favorable])**		
Birth weight (g)	**-48.96 (-79.15, -18.77)**	**0.002** [a]
Birth length (cm)	**-0.09 (-0.17, -0.02)**	**0.019** [a]
WHZ	**-0.09 (-0.17, -0.01)**	**0.045**
LAZ	**-0.05 (-0.09, -0.01)**	**0.019** [a]
BMIZ	**-0.1 (-0.18, -0.02)**	**0.015** [a]
WAZ	**-0.1 (-0.16, -0.03)**	**0.003** [a]
Apgar score at 1 minutes	0.02 (-0.03, 0.06)	0.446
**Moderate-CVH (vs low-CVH)**		
Birth weight (g)	**-228.47 (-441.46, -15.48)**	**0.036**
Birth length (cm)	**-0.62 (-1.18, -0.06)**	**0.029**
WHZ	-0.18 (-0.77, 0.42)	0.564
LAZ	**-0.33 (-0.63, -0.03)**	**0.029**
BMIZ	-0.3 (-0.89, 0.28)	0.303
WAZ	-0.42 (-0.86, 0.02)	0.064
Apgar score at 1 minutes	0 (-0.3, 0.31)	0.980
**High-CVH (vs low -CVH)**		
Birth weight (g)	**-266.19 (-489.64, -42.73)**	**0.020** [a]
Birth length (cm)	**-0.61 (-1.20, -0.02)**	**0.041**
WHZ	-0.28 (-0.91, 0.35)	0.384
LAZ	**-0.32 (-0.64, -0.01)**	**0.041**
BMIZ	-0.4 (-1.01, 0.21)	0.196
WAZ	**-0.48 (-0.94, -0.02)**	**0.041**
Apgar score at 1 minutes	0.1 (-0.23, 0.42)	0.556

The adjusted model covariates include maternal age, maternal education level, maternal parity, average income per month, maternal alcohol use before pregnancy, gestational weight gain, paternal age, gestational age, and infant sex.

[a] A false discovery rate less than 0.05, the bolded effect sizes indicate statistical significance ($P<0.05$).

Table. S4 The sensitivity analysis of adjusted association between maternal gestational CVH and birth physical growth, using gestational BMI at 24-32 gestation (instead of pre-pregnancy BMI) as the BMI metric (n=2445).

Gestational CVH exposure	β (95% CI) for birth physical growth	*P* value
**CVH score (per 10 points higher [more favorable])**		
Birth weight (g)	**-21.49 (-39.98, -3.00)**	**0.023** [a]
Birth length (cm)	-0.05 (-0.18,0.08)	0.461
WHZ	-0.03 (-0.09,0.02)	0.214
LAZ	-0.02 (-0.06,0.01)	0.161
BMIZ	-0.04 (-0.09,0.01)	0.085
WAZ	**-0.04 (-0.08,0.00)**	**0.033**
Apgar score at 1 minutes	-0.02 (-0.06,0.02)	0.400
**Moderate-CVH (vs low-CVH)**		
Birth weight (g)	-49.1 (-166.65,68.44)	0.413
Birth length (cm)	-0.62 (-1.18, -0.06)	0.353
WHZ	0.11 (-0.22,0.44)	0.519
LAZ	-0.19 (-0.39,0.02)	0.075
BMIZ	0.03 (-0.29,0.35)	0.847
WAZ	-0.09 (-0.33,0.16)	0.493
Apgar score at 1 minutes	0.01 (-0.22,0.25)	0.928
**High-CVH (vs low -CVH)**		
Birth weight (g)	-57.56 (-181.97,66.85)	0.364
Birth length (cm)	-0.48 (-1.37,0.41)	0.287
WHZ	0.01 (-0.34,0.36)	0.940
LAZ	-0.13 (-0.35,0.08)	0.229
BMIZ	-0.04 (-0.38,0.29)	0.800
WAZ	-0.1 (-0.37,0.16)	0.437
Apgar score at 1 minutes	0.02 (-0.23,0.27)	0.875

The adjusted model covariates include maternal age, maternal education level, maternal parity, average income per month, maternal alcohol use before pregnancy, pre-pregncancy BMI, paternal age, gestational age, and infant sex.

[a] The bolded effect sizes indicate statistical significance (*P*<0.05).

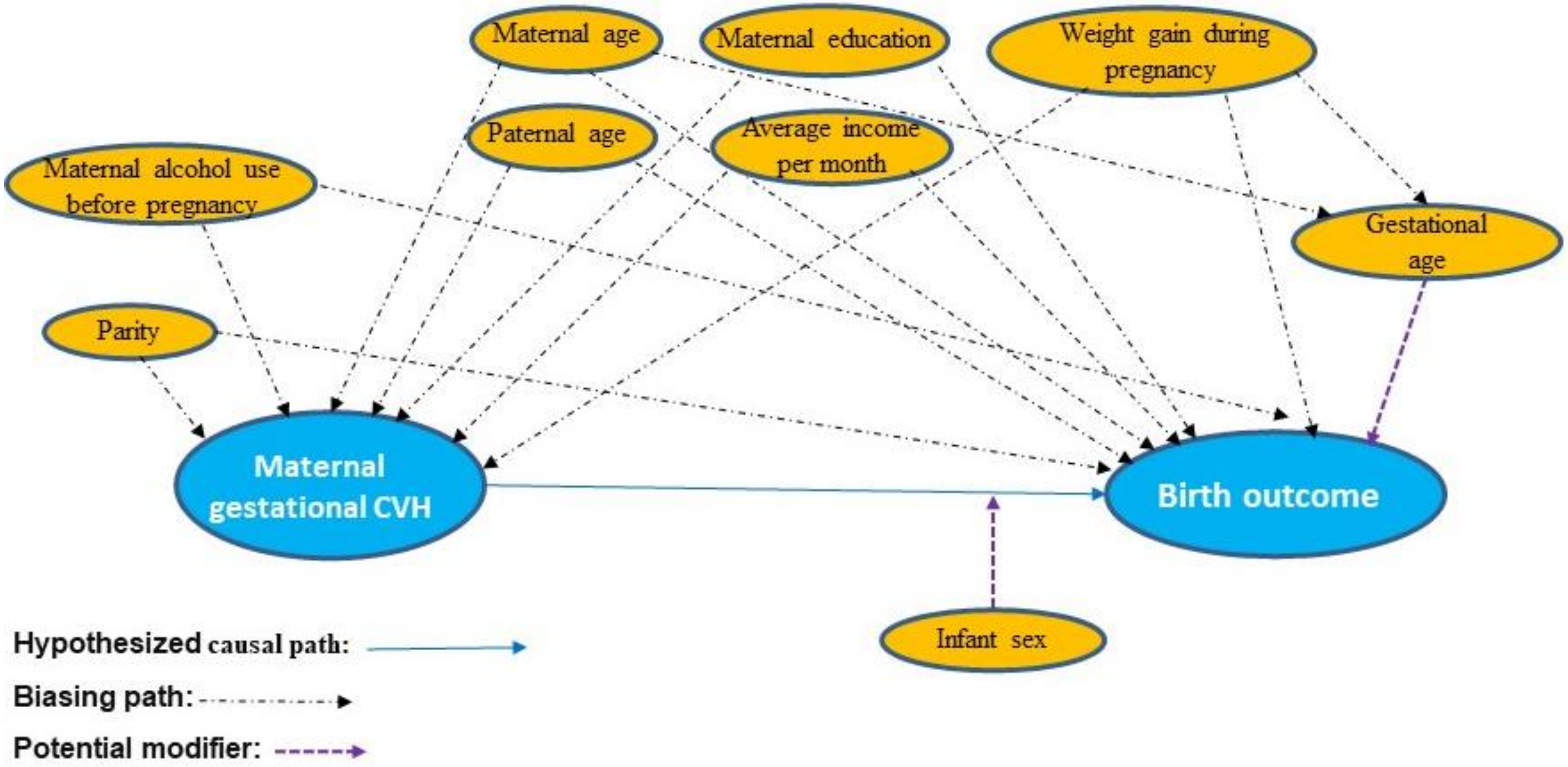

Figure. S 1 Directed acyclic graph depicting the relationship between maternal CVH in pregnancy and child birth outcome and relevant covariates available for this analysis.

Note: Blue ovals represent exposure and outcome; Yellow ovals represent the covariates and confounding factors; The blue dashed arrow represents the hypothesized causal path in this study; Black dashed arrows represent the biasing path; Purple dashed arrows represent the potential modifier factors. In order to maintain the consistency of the previous research, infant sex and gestational age as potential effect modifier factors were put into confounding covariates.

CVH metrics (Ideal status)	Preterm birth OR (95%CI)	SGA infant OR (95%CI)	LGA infant OR (95%CI)	LBW infant OR (95%CI)	Macrosomia infant OR (95%CI)
FPG	0.66 (0.43, 1.03)	1.41 (1.04, 1.90)	0.74 (0.60, 0.91)	0.89 (0.54, 1.44)	0.62 (0.45, 0.84)
TC	0.56 (0.36, 0.87)	0.93 (0.67, 1.28)	1.04 (0.83, 1.32)	0.62 (0.38, 1.03)	1.11 (0.78, 1.58)
BP	1.01 (0.65, 1.58)	1.16 (0.85, 1.59)	1.10 (0.89, 1.36)	1.12 (0.67, 1.86)	1.08 (0.79, 1.47)
Pre-pregnancy BMI	0.77 (0.46, 1.27)	1.66 (1.03, 2.66)	0.55 (0.44, 0.69)	1.09 (0.57, 2.08)	0.59 (0.43, 0.83)
PA	0.51 (0.07, 3.76)	0.26 (0.04, 1.94)	1.22 (0.63, 2.35)	——	0.43 (0.10, 1.81)
Sleep	0.99 (0.63, 1.55)	1.21 (0.87, 1.67)	0.92 (0.74, 1.13)	1.03 (0.62, 1.73)	0.92 (0.67, 1.26)
Diet quality	1.52 (0.96, 2.39)	0.80 (0.56, 1.15)	1.02 (0.81, 1.28)	1.00 (0.57, 1.75)	0.90 (0.63, 1.29)
Smking	1.71 (0.23, 13.03)	0.78 (0.27, 2.25)	1.00 (0.47, 2.12)	1.08 (0.15, 8.04)	0.63 (0.24, 1.64)

Figure. S2 Adjusted association between individual gestational cardiovascular health metrics and adverse birth outcomes.

Reference: non-ideal CVH metric;

All gestational metric (FBG, TC, BP, Pre-pregnancy BMI, PA, Sleep time, Diet quality, and smoking) were ideal metrics, and compared with non-ideal status.

The adjusted model covariates include maternal age, maternal education level, maternal parity, average income per month, maternal alcohol use before pregnancy, gestational weight gain, paternal age, and infant sex, as well as levels (ideal versus non-ideal [intermediate/poor]) of each of the other seven gestational CVH metrics.

The red squares represent statistical significance for rates of adverse pregnancy outcomes among maternal CVH metrics exposure.

"—" represents the absence of adverse birth outcomes in this CVH metrics.

Abbreviations: CVH, Cardiovascular health; FPG, fasting plasma glucose; TC, total cholesterol; BP, blood pressure; PA, physical activity; SGA, small for gestational age; LGA, Large for gestational age; LBW, low birth weight.

Gestational exposure	OR (95% CI)	*P* value
**CVH score (per 10 points higher [more favorable])**		
Preterm birth	0.74 (0.51, 1.08)	0.115
SGA	1.26 (0.90, 1.77)	0.173
LGA	0.81 (0.66, 0.99)	0.041
LBW	0.76 (0.44, 1.34)	0.349
Macrosomia	0.75 (0.64, 0.88)	0.001
**Moderate−CVH (vs low−CVH)**		
Preterm birth	0.61 (0.06, 5.73)	0.664
SGA	0.47 (0.05, 4.17)	0.495
LGA	0.38 (0.11, 1.29)	0.122
LBW	——	——
Macrosomia	0.43 (0.10, 1.73)	0.233
**High−CVH (vs Low−CVH)**		
Preterm birth	0.67 (0.06, 7.47)	0.748
SGA	0.47 (0.05, 4.64)	0.519
LGA	0.35 (0.09, 1.30)	0.116
LBW	——	——
Macrosomia	0.21 (0.04, 1.09)	0.064

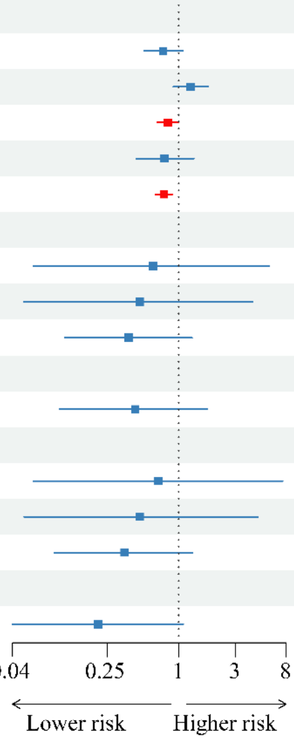

Figure. S3 The sensitivity analysis of adjusted association between maternal gestational CVH at 24-32 gestation and adverse birth outcome.

The adjusted model covariates include maternal age, maternal education level, maternal parity, average income per month, maternal alcohol use before pregnancy, gestational weight gain, paternal age, and infant sex.

The red squares represent statistical significance for rates of adverse birth outcomes among different maternal CVH exposure.

"—" represents the absence of LBW in high CVH.

Abbreviations: CVH, Cardiovascular Health, SGA, small for gestational age; LGA, Large for gestational age; LBW, low birth weight.

Gestational exposure	OR (95% CI)	*P* value
**CVH score (per 10 points higher [more favorable])**		
Preterm birth	0.84 (0.64, 1.11)	0.217
SGA	1.14 (0.93, 1.40)	0.139
LGA	0.93 (0.82, 1.06)	0.304
LBW	0.90 (0.66, 1.22)	0.491
Macrosomia	0.81 (0.67, 0.98)	0.030
1−minute Apgar score <7	0.84 (0.64, 1.11)	0.217
**Moderate−CVH (vs low−CVH)**		
Preterm birth	0.46 (0.13, 1.62)	0.226
SGA	1.18 (0.27, 5.12)	0.828
LGA	0.88 (0.42, 1.87)	0.746
LBW	0.48 (0.11, 2.15)	0.339
Macrosomia	1.32 (0.39, 4.48)	0.651
**High−CVH (vs Low−CVH)**		
Preterm birth	0.66 (0.17, 2.63)	0.557
SGA	1.67 (0.37, 7.57)	0.509
LGA	1.02 (0.46, 2.28)	0.956
LBW	0.54 (0.11, 2.75)	0.459
Macrosomia	1.24 (0.34, 4.60)	0.742

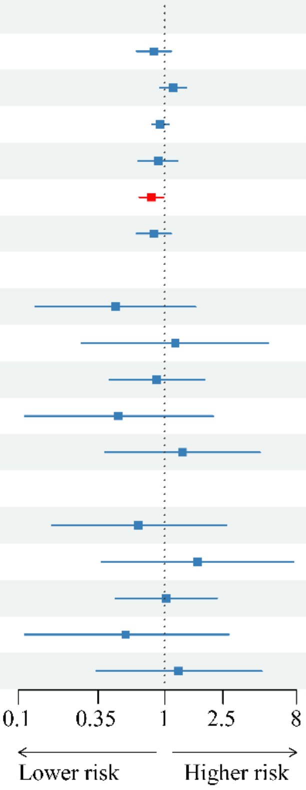

Figure. S4 The sensitivity analysis of adjusted association between maternal gestational CVH and adverse birth outcome, using gestational BMI at 24-32 gestation (instead of pre-pregnancy BMI) as the BMI metric.

The adjusted model covariates include maternal age, maternal education level, maternal parity, average income per month, maternal alcohol use before pregnancy, pre-pregnancy BMI, paternal age, and infant sex.

The red squares represent statistical significance for rates of adverse birth outcomes among different maternal CVH exposure.

Abbreviations: CVH, Cardiovascular Health, SGA, small for gestational age; LGA, Large for gestational age; LBW, low birth weight.

Affiliated Reference:

1 Zhao, L., Ma, G., Piao, J., Zhang, J., Yu, D., He, Y. *et al.* Scheme of the 2010-2012 chinese nutrition and health surveillance. *Zhonghua Yu Fang Yi Xue Za Zhi.* **50**, 204-207 (2016). https://doi.org/10.3760/cma.j.issn.0253-9624.2016.03.002

2 Wei, Q., Shi, H., Ma, X., Shi, Y., Zhang, Y. & Wang, L. The impact of maternal stress on offspring birth weight and the mediating effect of dietary patterns: The shanghai maternal-child pairs cohort study. *J Affect Disord.* **278**, 643-649 (2021). https://doi.org/10.1016/j.jad.2020.09.077

3 Gao, Y., Cui, L. F., Sun, Y. Y., Yang, W. H., Wang, J. R., Wu, S. L. & Gao, X. Adherence to the dietary approaches to stop hypertension diet and hyperuricemia: A cross-sectional study. *Arthritis Care Res (Hoboken).* **73**, 603-611 (2021). https://doi.org/10.1002/acr.24150

4 Lloyd-Jones, D. M., Allen, N. B., Anderson, C. A. M., Black, T., Brewer, L. C., Foraker, R. E. *et al.* Life's essential 8: Updating and enhancing the american heart association's construct of cardiovascular health: A presidential advisory from the american heart association. *Circulation.* **146**, e18-e43 (2022). https://doi.org/10.1161/CIR.0000000000001078

5 Macfarlane, D. J., Lee, C. C., Ho, E. Y., Chan, K. L. & Chan, D. T. Reliability and validity of the chinese version of ipaq (short, last 7 days). *J Sci Med Sport.* **10**, 45-51 (2007). https://doi.org/10.1016/j.jsams.2006.05.003

6 Syed, H., Slayman, T. & DuChene Thoma, K. Acog committee opinion no. 804: Physical activity and exercise during pregnancy and the postpartum period. *Obstetrics and gynecology.* **137**, 375-376 (2021). https://doi.org/10.1097/aog.0000000000004266

7 Shang, M., Lin, L., Ma, L. & Yin, L. Investigation on the suitability of the international association of diabetes and pregnancy study group diagnostic criteria for gestational diabetes mellitus in china. *Journal of obstetrics and gynaecology : the journal of the Institute of Obstetrics and Gynaecology.* **34**, 141-145 (2014). https://doi.org/10.3109/01443615.2013.832177

8 Zhang, H., Li, Y., Zhao, X., Mao, Z., Abdulai, T., Liu, X. *et al.* The association between psqi score and hypertension in a chinese rural population: The henan rural cohort study. *Sleep medicine.* **58**, 27-34 (2019). https://doi.org/10.1016/j.sleep.2019.03.001

9 Chen, C. & Lu, F. C. The guidelines for prevention and control of overweight and obesity in chinese adults. *Biomedical and environmental sciences : BES.* **17 Suppl**, 1-36 (2004).

10 Perak, A. M., Lancki, N., Kuang, A., Labarthe, D. R., Allen, N. B., Shah, S. H. *et al.* Associations of gestational cardiovascular health with pregnancy outcomes: The hyperglycemia and adverse pregnancy outcome study. *Am J Obstet Gynecol.* **224**, 210 e211-210 e217 (2021). https://doi.org/10.1016/j.ajog.2020.07.053

11 Xie, X. & Gou, W. *Obstetrics and gynecology*. (8th Ed, 2013).

12 Hypertensive Disorders in Pregnancy, S. o. O. a. G., Chinese Medical Association. Chinese expert consensus on blood pressure management during pregnancy (2021). *Chinese Journal of Obstetrics and Gynecology.* **56**, 737-745 (2021).

13 Khan, S. S., Cameron, N. A. & Lindley, K. J. Pregnancy as an early cardiovascular moment: Peripartum cardiovascular health. *Circulation Research.* **132**, 1584-1606 (2023). https://doi.org/10.1161/circresaha.123.322001

14 Department of Disease Control, M. o. H., People's Republic of China. *Guidelines for the prevention and control of overweight and obesity in chinese adults*. (People's Medical Publishing House, 2003).

15 Lloyd-Jones, D. M., Hong, Y., Labarthe, D., Mozaffarian, D., Appel, L. J., Van Horn, L. *et al.* Defining and setting national goals for cardiovascular health promotion and disease reduction: The american heart association's strategic impact goal through 2020 and beyond. *Circulation.* **121**, 586-613 (2010). https://doi.org/10.1161/CIRCULATIONAHA.109.192703

16 Perak, A. M., Lancki, N., Kuang, A., Labarthe, D. R., Allen, N. B., Shah, S. H. *et al.* Associations of maternal cardiovascular health in pregnancy with offspring cardiovascular health in early adolescence. *JAMA.* **325**, 658-668 (2021). https://doi.org/10.1001/jama.2021.0247
